# The Impact of the COVID-19 Pandemic on Patients from a Bariatric Program: A Qualitative Analysis of Their Perceptions of Health and Well-Being

**DOI:** 10.3390/healthcare10050780

**Published:** 2022-04-22

**Authors:** Jennifer M. Klasen, Deborah M. Tynes, Caspar J. Peterson, Romano Schneider, Katharina Timper, Ralph Peterli, Cameron L. Randall, Tarik Delko

**Affiliations:** 1 Clarunis, Department of Visceral Surgery, University Center for Gastrointestinal and Liver Diseases, St. Claraspital and University Hospital Basel, 4002 Basel, Switzerland; casparjoyce.peterson@clarunis.ch (C.J.P.); romano.schneider@clarunis.ch (R.S.); ralph.peterli@clarunis.ch (R.P.); tarik.delko@clarunis.ch (T.D.); 2 Clinic of Endocrinology, Diabetes and Metabolism, Department of Clinical Research, University Hospital Basel, 4031 Basel, Switzerland; deborah.tynes@stud.unibas.ch; 3 Division of Endocrinology, Diabetes and Metabolism, University Hospital Basel, 4031 Basel, Switzerland; katharina.timper@usb.ch; 4 Department of Oral Health Sciences, School of Dentistry, University of Washington, Seattle, WA 98195, USA; clr333@uw.edu

**Keywords:** qualitative study, patients with overweight, patients with obesity, bariatric patients, well-being, grief, COVID-19 pandemic, COVID-19

## Abstract

Introduction: The study was conducted to explore the perceptions of patients from a bariatric program who have undergone or will undergo bariatric surgery during the ongoing COVID-19 pandemic, specifically as related to their struggles with health issues and their psychological well-being. Materials and Methods: We conducted semi-structured, in-depth interviews with nineteen pre- or post-bariatric patients to generate data on their perceptions of COVID-19. Consistent with the methods of constructivist grounded theory, we collected and analyzed data iteratively through a constant comparative process for data coding and develop themes in the transcripts. Results: We identified themes to summarize the pandemic-associated experiences of our cohort as follows: their life structure before COVID-19, the turning point with changes and adaptations, and the impact of isolation on psychological well-being. We identified grief due to loss of social contacts as well as physical and psychological health impairment as consequences of pandemic-related lifestyle changes. Most participants were not aware of overweight and obesity being major risk factors for worse outcomes of COVID-19. We developed a theme-based theory on patients’ perceptions and fears regarding the pandemic as they live through phases of grief. Discussion: Most participants shared critical perceptions about their own somatic and psychological health. These findings may inform recommendations and strategies for both patients and healthcare professionals to manage the challenges potentially presented by this vulnerable patient group in the context of the COVID-19 pandemic.

## 1. Introduction

COVID-19 was officially declared a pandemic by the World Health Organization (WHO) on 11 March 2020 [1]. On 16 March 2020, Switzerland tightened security measures and announced a state of emergency, shutting down all businesses and services deemed non-essential for a period of 6 weeks until 26 April 2020 [2]. This shutdown forced hospitals to cancel non-essential elective surgical procedures and outpatient consultations for all non-emergency cases, including those in the field of bariatric surgery.

In the background of the COVID-19 pandemic is an ongoing and decades-long pandemic of obesity and metabolic syndrome, which the WHO has termed the “global obesity epidemic” [3]. The incidence of metabolic syndrome is increasing worldwide, rooted in a combination of obesity, metabolic imbalances such as glucose intolerance, insulin resistance, dyslipidemia, and hypertension [4,5]. Bariatric surgery is the most effective treatment for morbid obesity, with durable weight loss and control of obesity-related comorbidities demonstrated in randomized controlled trials [6,7,8,9,10]. 

Overweight and obesity were identified as well-known risk factors for severe outcomes of COVID-19 through inflammatory and immunological mechanisms [11,12]. Patients with a body mass index (BMI) of 25 kg/m^2^ or higher are at increased risk of hospitalization, need for mechanical ventilation, and death [13,14], and related comorbidities have been correlated with a severe clinical course of COVID-19 and increased mortality [12,15,16,17,18]. Therefore, physicians and patients should be aware that both overweight and obesity create significant vulnerability for COVID-19 infection and elevate the risk of an unfavorable outcome [11,19,20,21].

Patients with a history of overweight or obesity may experience feelings of isolation and stigmatization associated with their weight [22]. In addition to being psychologically distressing, social isolation and loneliness are associated with increased mortality risk [22]. Moreover, both isolation and weight stigma are associated with anxiety, depression, substance use, and poor general mental health [23,24,25]. During the COVID-19 pandemic, social interactions have been further reduced due to the imposed shutdown measures and social distancing requirements [2]. It is thus crucial to explore how these patients experience and deal with social isolation and what their major difficulties are.

The objective of the present study was to explore perceptions in regard to the COVID-19 pandemic and related challenges on bariatric patients. We aimed to explore individual experiences with the COVID-19 pandemic, focusing on quality of life before the pandemic, beliefs about comorbidities and health, and well-being during the shutdown.

## 2. Materials and Methods

### 2.1. Study Design

We conducted a qualitative study using constructivist grounded theory, which seeks to understand underlying psychological processes within a specific context [26]. Consistent with that approach, we purposely recruited [27]—in collaboration with our interdisciplinary and interprofessional team of physicians and nutritionists—patients whom we deemed to be thoughtful about their weight and health situation, especially influenced by the pandemic, and who expressed interest in study participation.

The local ethics committee approved the study (Ethikkommission Nordwest- und Zentralschweiz 2020-01178). The study was conducted in adherence to the Consolidated Criteria for Reporting Qualitative Research (COREQ) guidelines.

### 2.2. Participants, Recruitment, and Data Collection

We recruited patients of different ages and genders who had either undergone or were scheduled to undergo bariatric surgery, were overweight (BMI over 25 kg/m^2^ as reference for a worse COVID-19 outcome) at the time of recruitment and were sufficiently proficient in German or English. Aiming to obtain a variety of viewpoints, we included patients from our bariatric program who were over 18 years old and were at different stages of their bariatric journeys (pre-, peri-, or postoperative). Patients who fulfilled the inclusion criteria were informed by one of their healthcare providers about our study and its purpose during medical consultations (J.K., T.D., and a team of endocrinologists (K.T.)) or nutritional counseling sessions.

Patients who showed interest were called by the interviewer (D.T.) and given more detailed information about the study’s objective, structure, and procedure. After a patient’s verbal commitment, the interviewer obtained written informed consent from them. We sent a semi-structured, in-depth interview guide to the patients beforehand to reduce potential stress and anxiety. Participants were encouraged to contact the research team at any time to address questions or uncertainties about the study documents. Additionally, we offered psychological support from our research team in the event that interview questions triggered unpleasant emotions. We informed all patients that their participation was voluntary and confidential, and they could at any point choose not to answer a question, interrupt or discontinue the interview, or later on withdraw from the study.

So that participants would be comfortable, we let them choose their preferred interview setting within the restrictions imposed by the ongoing COVID-19 pandemic, respecting their individual concerns. Three participants insisted on an individual face-to-face interview, following the official guidelines on maintaining distance and wearing face masks. Three participants agreed to a Zoom video call, which allowed the interviewer to observe non-verbal communication, which she documented in her field notes. Most participants (*n* = 13) chose a telephone interview. Depending on participants’ individual desire to disclose their narratives during the semi-structured interview, the duration of the interview varied from 30 to 95 min. For logistical reasons, the interviews were conducted in two phases: phase 1 (interviews P1–P10) between 20 July and 30 July 2020 and phase 2 (P11–P19) from 22 September to 13 October 2020.

The first two interviews were pilots; however, they were of sufficiently high quality to be included in the data set with the participants’ consent. All interviews were audio-recorded, transcribed verbatim, and assigned non-defining codes for anonymization upfront. Nineteen participants comprised a sufficient sample, meaning that no changes to the themes had to be made based on the interviews, while 21 patients agreed to participate in the study [28].

### 2.3. Data Analysis, and Reflexivity of the Research Team

For data analysis, JK and DT jointly developed the initial codes in regular meetings. During these meetings, they discussed further data collection and elaborated possible adjustments while identifying relevant themes. The analysis of all transcripts was performed iteratively and collaboratively following the method of grounded theory [26]. In a constant comparative approach, further data collection and analysis was strengthened by new interview transcripts [27,29]. All themes were presented to the research group, and we developed the theory of grief we present later in this paper. For transcript coding, we used the software Quirkos in order to create a visual representation and develop an understanding of the connections of the interview data.

JK the principal investigator of this study, is a bariatric surgeon with qualitative research expertise and the thesis supervisor of DT, who herself is a first-year resident. DT studied the basics of bariatrics and its interdisciplinary setting, observed bariatric operations, and attended different interdisciplinary group meetings to gain insight into the several steps a patient with obesity has to go through before surgery. The broader research team included two junior bariatric surgeons (RS and CP), two senior surgeons who lead the bariatric program (RP and TD), an endocrinologist with extensive experience of treating patients with obesity (KT), and a clinical health psychologist with expertise in the interaction of physical and mental health (CR). JK. RS, CP, RP, TD, and KT represent a team of physicians who advocate for people with overweight and obesity and understand their daily struggles and challenges. Our research team speaks out against the stigmatization of increased body weight, and all of us engage in obesity research to optimize the treatment and management of this disease. Our experiences as doctors and researchers, aware of patients’ often longstanding histories of physical or psychological issues related to overweight or obesity, have impacted the study. Additionally, we realized that our individual experiences confronting the simultaneous challenges of the COVID-19 pandemic might influence our interpretation of the collected data.

## 3. Results

We interviewed 12 women and 7 men with overweight or obesity between 24 and 61 years of age. Fifteen participants were in pre-, peri-, or postoperative bariatric surveillance, while 4 were undergoing conservative treatment. Their BMI ranged between 27.8 and 55.4 kg/m^2^; 14 participants gained weight during the first pandemic wave, 4 lost weight, and 1 showed stable weight (Table 1). Below, we illustrate the major themes with supporting quotes from our participants, presenting all themes with additional quotes in Table 2 (Table 2).

### 3.1. Life Structure before the Pandemic and Emergence of COVID-19

Before the COVID-19 pandemic, most participants had a structured daily life: a job, regular physical activity (e.g., bicycling, swimming, football, walking), and traditional social interactions with family and friends. One participant reported: “At weekends, we were often on the road and also spent a lot of time outside. Or we met friends….” (P2). A few participants, however, described being socially isolated and minimally active before COVID-19 and thus did not experience a noteworthy change (P5, P18). One participant explained: “… We don’t have friends because we are focused on each other [the participant and his wife]” (P9). However, most participants described an individually experienced turning point when realizing that COVID-19 would change daily life: “I think you only really realized it when everything was closed. The restaurants, the clubs, and then I was afraid of going out because you didn’t know where you might get infected and how” (P1). Others realized the severity of the situation when major local events were canceled.

“That was on Friday before the local carnival. As the carnival was canceled, I realized that we are dealing with a little more than an ordinary flu wave. That was the moment for me. That’s when I realized that it is serious.” (P6).

After experiencing individual-specific turning points, participants were forced into a new life with adjusted structure.

### 3.2. Changes and Adaptations

Every participant described experiencing troubling situations during the shutdown. They adapted to new life circumstances such as the closing of borders, not being able to see family members and friends, the loss of their job and resulting financial issues, and even mourning the death of a family member while not being able to attend their funeral. One participant recalled: “The greatest difficulty I had staying at home was not having contact with my family. We only communicated by telephone. That was the most difficult adjustment” (P2).

All participants had to change their routines, describing the most significant restrictions on activities as being triggered by uncertainty due to COVID-19.

Participants struggling with obesity should follow individual nutritional guidelines. For the sake of wider variety and cost savings, some of our participants regularly drove to grocery stores in Germany and France before the pandemic. The closure of borders forced these participants to change their shopping habits. The amount of money they were required to spend rose substantially, which was financially challenging. For one participant, this proved to be the greatest problem: “It (the most difficult adjustment) was the closing of the borders. That was very difficult for me. At the time, my financial situation was not strong, which was difficult. And life in Germany is cheaper, and normally we got groceries in Germany [instead of Switzerland]” (P13). Regarding fear of SARS-CoV-2 infection, two patients had food delivered to their homes (P3, P18), while one reported that going to fetch groceries led to anxiety: “The first time I went shopping again, it was only at 8 p.m. with heart palpitations” (P3). In the end, a limited choice of food combined with financial issues influenced the health of these patients because their diet no longer completely fulfilled their needs.

Further adaptation was the result of restriction of social contacts and increased communication via electronic devices. Some participants reported that video phone calls and text messages became increasingly frequent due to the shutdown situation, which sometimes had a negative impact on well-being: “I had the most trouble with staying at home and breaking off contact with my family. That everything was only by phone. That was the hardest adjustment for me” (P2). However, the shutdown and associated social isolation were experienced differently by individual participants: some reflected on having mixed feeling about staying at home, while others felt alone, even more isolated and distanced in society.

“I experienced the time [shutdown] as a gift for myself and my family… But I am also very stressed because I have a great fear for my family. My children wanted to go outside, but I told them that they have to stay inside.” (P4).

Social distancing and the fears described above influenced the psychological health of most participants.

### 3.3. Emotional Well-Being, Fear, and Grief

The COVID-19 pandemic’s social and emotional impact was particularly salient for all participants. Social distancing resulted in staying home alone, leading to social isolation. These experiences were described by some as comfortable, because there was no need to explain why they were staying home and not participating in social events, for example. Participants living alone felt, in general, more isolated, and lonelier than before (P8, P18).

Overall, the pandemic had a negative psychological impact on all participants, who described emotions such as fear, anxiety, grief, depression, frustration, anger, and loneliness. One participant reported:

“I did it (coped) by eating. Which caused even more stress for me because I knew the surgery was coming. And since October 2019 I was slowly losing weight. But when school was cancelled from February to April, I gained 15 pounds, which is around 7.5 kg, in 3 months. Which even caused more stress for me. I am already on antidepressant medication.” (P8).

Further, she expressed feelings of loneliness and depression:

“So, I was very, very lonely, very, very depressed. I’m still feeling like I’m going through depression. It’s getting a little better, but it’s hard. I am very isolated here. I don’t have any family here. And through COVID, everything was shut down, and there was no interaction except maybe by phone with family.” (P8).

Participants described a change in their emotions over time, comparable to the stages of grief: From denial and anger, through bargaining and depression, to eventual acceptance [30]. At the pandemic’s outset, the predominant emotion was fear. When a shutdown was imposed in Switzerland from 16 March to 19 April 2020, and the number of cases gradually decreased again, one participant experienced a reduction of fear: “At the beginning I was more afraid of being infected. Now, I think that if you follow the hygiene rules, you are less at risk. So the fear is no longer as great as it was at the beginning” (P1). One participant reflected on her emotions by referencing the stages of grief:

“To be honest, I am a little bit angry towards corona, actually very angry. But I am angry with the people. First, it seemed a bit ridiculous and then I was actually scared… In the beginning it was a bit like the stages of grief, you could almost say. It was denial or compromise or just fear and now it’s like everyday life. It’s like normal, but it’s just annoying and tedious and we are restricted. We are not restricted in everything, but it is also annoying because I am aware that it will take at least another 2 years until it is really gone. And nobody knows for sure if they will really find a vaccine against it.” (P14).

All participants experienced some degree of emotional impact such as: “I grieved not being allowed to talk to people in person and not being able to see them, and I balanced the frustration with comfort eating” (P1). Ultimately, most patients agreed on the same future wishes: a better understanding of the viral disease, the development of a vaccine, and a daily life structure similar to pre-pandemic times.

Overall, stress and the degree of impact on psychological well-being increased as a function of social distancing and isolation, triggering emotional eating in some patients. However, participants also grappled with other health issues, such as exacerbation of existing obesity due to weight gain triggered by less strict eating habits.

### 3.4. Perspectives on Health Issues and Risk of COVID-19 Infection

The shutdown led to a substantial reduction in physical activity for many participants, and most experienced weight gain. One participant explained: “I just do nothing at all anymore and watch a lot of TV and eat a lot… I have gained 11 kg since corona” (P14).

Through weight gain, patients with obesity expected an even higher risk of experiencing a severe course of COVID-19 [12,20,30,31]. One participant counted herself in the high-risk group, acknowledging obesity as a risk: “Yes, because I have many heart issues I am at risk. Also, because of my obesity. And, I am under immunosuppression through chemotherapy” (P5). However, most participants described the comorbidities of obesity, such as hypertension or diabetes mellitus, as risk factors for COVID-19 infection, but not the obesity itself. This was the case especially for younger patients, one of whom said: “No, actually I don’t see myself as part of the risk group… I think most of them are just older people, and I think I’m still young. I think as long as I stick to everything that is recommended, I am good” (P13). Some participants recognized no risk at all, although they were aware of their existing obesity: “No, basically not [a risk patient], because I am healthy and have no previous health issues. Well, I am overweight, but otherwise, I don’t have any previous conditions” (P2).

In addition, participants perceived a lack of communication between different healthcare providers, resulting in contradictory or confusing information:

“At the beginning, they [family doctor] said I don’t need a confirmation for work that I belong a risk group [for COVID]. However, 2 weeks later, my office said I need one to define me as a patient at risk. Then, I thought that I would definitely get it [the confirmation] through my family doctor. But the medical nurse told me she would not categorize me as a high-risk patient, and she doesn’t know why she should write this letter. So, I explained her that I have high blood pressure … And then she said I can get it through the University Hospital. In the end, I finally got it from my nephrologist. She confirmed that I was a high-risk patient.” (P7).

Three participants described a lack of support from primary care physicians, prompting a switch to another doctor in two of the three.

## 4. Discussion

This qualitative study involved in-depth exploration of the perceptions of bariatric patients regarding potential physical and mental health issues associated with the COVID-19 pandemic. We summarized these perceptions into four main themes: the life structure before the pandemic and emergence of COVID-19; changes and adaptations; emotional well-being, fear, and grief; and perspectives on health issues. Most patients described significant effects on their somatic and psychological health. The strongest negative impacts on well-being were caused by the shutdown, isolation with reduced social contacts and less structured daily life activities, financial problems, or fear of becoming infected. Those external and internal stressors may have led to emotionally triggered weight gain and additional stress and depression. Our study participants appeared only vaguely aware that obesity itself is a risk factor for COVID-19 infection and poor outcomes.

Patients suffering from obesity are considered at high risk of a worse outcome following COVID-19 infection [32,33,34]. The COVID-19 pandemic introduced or exacerbated our patients’ social isolation, which had emotional and physical implications. Other studies demonstrated that working from home, staying indoors, and isolation at home led to physical inactivity, increased snacking, and emotional eating [35,36,37]. These behaviors were associated with internal stress, resulting in depressed mood, anxiety, and intensification of disordered eating habits [36,37,38]. Staying at home and being apart from family and friends, loss of employment and the resulting financial issues, limited food accessibility due to the closure of borders, and less physical activity represented the major structural life changes.

Exploring our participants’ difficulties during the COVID-19 pandemic and the emotional implications thereof, we identified an impact on well-being which was strongly influenced by social distancing, being apart from loved ones, and isolation. Among our participants, we identified the main stressors during isolation as: fear of infection; frustration about defenselessness; anxiety regarding the pandemic’s implications, resulting in snacking, boredom, or emotional eating; post-isolation factors such as job loss with financial insecurity; and weight gain causing even more inactivity. Other studies have drawn similar conclusions [39,40,41,42,43]. Internal emotional stress increased as a function of isolation, and existing coping strategies often seemed insufficient for dealing with the new emotional circumstances, leading to loneliness, anxiety, depressed mood, or anger.

**Figure 1:**
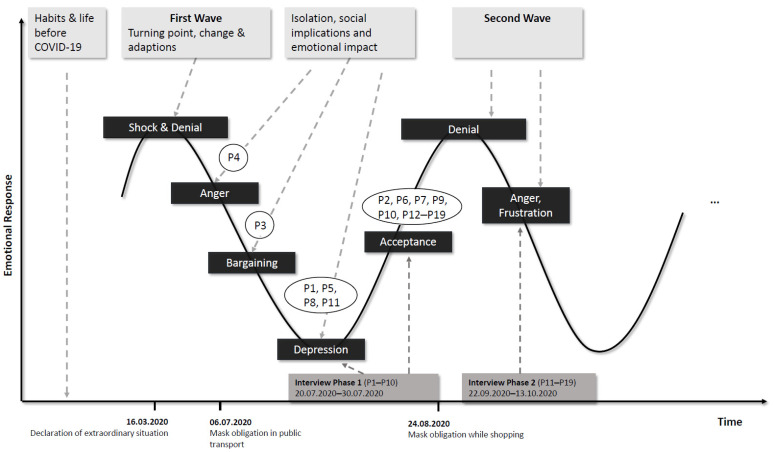
Participants’ stages of grief.

During the ongoing COVID-19 pandemic, our participants seemed to pass through the successive stages of grief, a model introduced by Kübler-Ross in 1969 [30,44,45]. These five stages, as adapted by David Kessler, are: shock and denial (“this virus doesn’t existing and won’t affect us”); anger (“this virus is taking away our life structure and making us be isolated at home”); bargaining (“if I isolate myself then after the shutdown everything will be okay again”); depression (“I am so limited in my everyday life, I feel depressed”); and acceptance (“this virus is with us to stay and we have to adapt our way of living”) [46]. Research has shown that individuals experience various negative feelings—e.g., denial, anger, fear, and helplessness—especially at the beginning of unprecedented circumstances such as a pandemic [46,47,48]. Although individual participants may have been at different stages and at different points of their bariatric journey at the time of their interview, most seemed to have reached the stage of acceptance over time (Figure 1). After a phase of anxiety and anger about the forced changes, the stage of bargaining was expressed as accepting the risk of infection and mandatory social isolation. At the same time, participants were optimistic that everything would be “normal” again after some time. Because the pandemic did not come to an end, and restrictions and isolation had to be continued, some participants were drifting into the stage of depression. The stage of acceptance, classified as facing the current situation and accepting its inevitability, is most likely affecting everyone’s behavior during the ongoing COVID-19 pandemic.

Now, during new waves of the pandemic, with dramatic increases in infections worldwide, some of our participants seem to have developed acceptance about the virus. Other participants described anger and frustration about what might come, fearing further limitations or another shutdown. Given the findings of previous research, the presence of all these negative feelings has the potential to trigger emotional eating behavior [49]. Moreover, we can imagine that the ongoing COVID-19 pandemic has led to longer waiting times for bariatric surgery, impaired access to medication, and a greater use of telehealth to maintain a sufficient level of healthcare provision to this vulnerable patient group. Related research has led to similar findings [50,51].

A broad range of studies have shown that COVID-19 increases the morbidity and mortality of persons with BMI > 25 kg/m^2^ [17,20,34,52,53]. In those studies, most participants struggled with emotional eating and decreased physical activity, and gaining weight [17,20,34,52,53]. In our study, most participants knew that hypertension and diabetes mellitus were risk factors for a severe course of COVID-19 but were not aware that overweight or obesity itself is a risk factor. In Switzerland, 42% of the adult population is overweight, which is in the range of other European countries (about 40–60%), and about 11% of Swiss adults are obese [54,55,56]. There may be different reasons why overweight and obesity were not understood as a risk factor by our study population. On the one hand, being overweight may be normalized if almost half of the overall population is overweight and may thus not be perceived as a risk factor for health issues associated with the COVID-19. On the other hand, the stigmatization of obesity may have played a role, making even healthcare providers reluctant to talk about this issue. Additionally, the information flow and load during the COVID-19 pandemic was overwhelming for both patients, and healthcare providers.

Additionally, from the perspective of our participants, some primary care physicians were also unaware that patients with increased bodyweight are at higher risk. All healthcare professionals should appreciate the importance of increased bodyweight and related comorbidities as risk factors [21,57,58,59] for their patients. Our findings suggest that we need to take steps to ensure overall awareness that patients with increased bodyweight are potentially more vulnerable to infection and also more contagious (due to a defective immune system), and that these patients depend on close monitoring and collaborative care within a functioning healthcare system [60,61] These important considerations seem not to have been efficiently conveyed (or implemented) during the ongoing COVID-19 pandemic. Additionally, and relatedly, in view of the risk of COVID-19 infection and the danger of a severe outcome we advocate keeping all medical disciplines operational, including conservative and surgical treatment of obesity. We must adapt our healthcare systems in light of the experience gained during the first wave of the COVID-19 pandemic and do everything feasible to support the most vulnerable groups in the best possible way [19]. Furthermore, we should act in an interdisciplinary fashion to increase our patients’ knowledge of COVID-19 and the related risks, including supporting those who may already have been struggling by ensuring more straightforward access to healthcare and consistent health messaging. Accordingly, we have introduced an educational segment about the potential consequences of COVID-19 to the group lectures we offer our patients before surgery and have started to raise awareness of the study results in our dialogue with patients and colleagues.

## 5. Limitations

First, based on our sampling strategy, we recruited patients in different consultation settings; this resulted in interviewing a diverse cohort of participants with a bariatric background, including pre-, peri-, and postoperative patients as well as patients receiving conservative treatment for overweight or obesity. Such a wide-ranging group includes people with different medical and life situations and problems, most likely with different expectations from their recent or upcoming surgery or conservative treatment, especially in the context of the pandemic. Second, we conducted the interviews in two different phases during the ongoing COVID-19 pandemic, which probably introduced systematic variability in participants’ narratives. On the other hand, such an interview strategy offered some insight into individuals’ problems and challenges at different times during the pandemic. Third, the data analysis and interpretation were carried out in German; the presentation of results in English may introduce a translation and interpretation bias. Eighteen of the nineteen interviews were conducted in German, one in English, by a native speaker of German (DT). Finally, due to the COVID-19 pandemic we conducted a single-center rather than a multicenter study.

## 6. Conclusions

This qualitative study identifies a significant impact of the COVID-19 pandemic on the daily life structure and well-being of patients with a bariatric background, resulting in weight gain and psychological distress. Social distancing leads to isolation and associated emotional stress in patients who are often already psychologically vulnerable. The results suggest that during and soon after the ongoing COVID-10 pandemic it may be necessary to provide emotional support and psychological treatment to patients in a bariatric program. Although obesity seems to lead to worse COVID-19 outcomes, this study showed that patients with present or previous obesity associated their increased risks with their comorbidities rather than with obesity or overweight itself. Regarding their health issues and risk situations, patients need education on the consequences of belonging to a high-risk group. Expanding all healthcare professionals’ understanding of these issues may be critical for early identification of psychological stress reactions and the provision of emotional well-being and health behavior support, especially for patients with obesity. Moreover, we anticipate that our patients are experiencing restrictions due to the pandemic itself similar to those experienced by the non-obese population [42,43]. Overall, it is important to remember that patients with obesity are more vulnerable to a more severe outcome of COVID-19 infection [13,34]. Further studies should evaluate the long-term effect of pandemic circumstances on the odds of moderate to severe psychological consequences for individuals, especially those at increased risk such as patients with overweight and obesity. In this regard, future research could unfold in two ways. First, exploring why individuals may not realize that overweight and obesity are risk factors for COVID-19 and other diseases may yield insights into their perceptions of their own health. Second, how they might make up for the somatic and psychological burden of COVID-19 and how we as healthcare providers could offer our support.

## Figures and Tables

**Table 1:** Participants’ demographics (anonymized through expression of age as a range, weight in terms of BMI, and weight loss as a percentage).

Participant	Sex	Age (Years)	Bariatric Treatment (Year of Surgery, Drug)	Other Diagnoses	BMI (kg/m^2^)	Total Weight Loss (%)	Weight Change during COVID-19 (%)
1	F	44–46	LRYGB (2000), LRYGB (2005), BPD/DS (2014), treated with GLP-1 analog (2020)	Smoking history	47.5	−11	−10 (With GLP−1 analog)
2	M	33–35	LRYGB (2018)	HBP, currently without medication	28.6	−28	+4.9
3	F	51–53	Conservative	Arterial fibrillation, HBP, asthma, OSAS with CPAP	43.6	−7	+3.9
4	F	42–44	SG (2017)	None	28.4	−19	+5.6
5	F	53–55	SG (2015)	OSAS, HBP	27.8	−27	+6.9
6	M	34–36	Conservative	Renal insufficiency St 3b A2–3, HBP	29.2	−16	+4.1
7	F	50–52	Preoperative LRYGB (planned 2020)	HBP, pre-T2D, smoking history, COPD GOLD stage 2, severe OSAS	32.4	−10 (Presurgical diet)	−11.7
							(Presurgical diet)
8	F	41–43	LRYGB (2020)	Pre-T2D, OSAS with CPAP	53.4	0	+5.5
9	F	54–56	SG (2017)	HBP, OSAS with CPAP, pre-T2D	46	−25	+4.8
10	F	55–57	LRYGB (2013), BPD/DS (2015)	None	33	−50	+4.4
11	F	22–24	LRYGB (2020)	HBP without medication, allergic asthma	42.9	0	−9.0
							(Shortly after surgery)
12	M	40–42	LRYGB (2011)	T2D, HBP, OSAS	44.7	−10	0
13	F	35–37	Preoperative for LRYGB (planned 10/2020)	None	45.2	−8	+0.9
						(Presurgical diet)	
14	F	25–27	Preoperative for SG (planned spring 2021)	HBP, Pre-T2D	46.3	−29	+0.8
15	M	52–54	Conservative	T2D	31.1	−7	+2.8
16	F	28–30	Preoperative for LRYGB (planned 11/2020)	None	41	−6	+2.7
17	M	35–37	Preoperative for LRYGB (planned 12/2020)	HBP	41.7	−6	+7.4
18	M	59–61	LRYGB (09//2020)	Pre-T2D, HBP, OSAS, COPD GOLD stage 2	47	−13	−2.8
							(Shortly after surgery)
19	M	53–55	Conservative with drug support with GLP−1 analog (2020)	OSAS, HBP, T2D	35.6	−6	+4.2

LRYGB: Roux-en-Y gastric bypass, BPD/DS: biliopancreatic diversion with duodenal switch, SG: sleeve gastrectomy, HBP: high blood pressure, OSAS: obstructive sleep apnea syndrome, CPAP: continuous positive airway pressure, COPD: chronic obstructive pulmonary disease, T2D: type 2 diabetes.

**Table 2:** Master table of themes and supporting quotations.

Major Themes	Supporting Quotations and Minor Themes
Life structure before the pandemic and emergence of COVID-19	“It was not very different before and after COVID. I got up at about 7.00 a.m. and went for a walk with my dog. Then I did the household, at my own pace because of the accident.” (P10) “I always got up at 5.30 a.m. with my boyfriend, and then I took the bicycle to work… I enjoyed work, also because of the social contacts there… I don’t have many social contacts otherwise. I have one close friend, and we meet once a month.” (P13) Social contacts: “We don’t have friends we hang out with. Some of them have already died, and we are more concerned about ourselves… And we did not often go to a restaurant anyway.” (P18)
Changes and adaptations	Groceries and financial issues: “All that time, we went for groceries only twice, and only my husband. Before the lockdown, I bought a lot of food, everything for about three months and then we had enough at home.” (P4) “And it was also difficult with the food because I suddenly had much less money. We couldn’t shop over the border in Germany, and the budget was quite tight. And then the food was no longer ideal for my needs.” (P2) Social contacts and activities: “I reduced all social contacts. And I had my food delivered until June… And when I go to Claraplatz or go shopping somewhere, I get very annoyed by people just standing around or strolling through the streets.” (P3) “Sport at the moment, not yet. Because at soccer training there are still too many people. But actually, I would like to start there again slowly in August. But now that I see the numbers, I have to think it over again. On the other hand, I noticed how it would be good for me doing more sports again.” (P6) “The telephone contact was okay for me. But I still have a daughter who is 22 years old. And she has now taken her apartment in January; it’s the first time. And I also looked after her a little bit. And because of the whole corona situation, I couldn’t visit her. But for her, it was just as bad as for me.” (P7) “I spent a lot of time sitting at home alone or going for a walk outside. Communication with my children and my wife also collapsed relatively quickly. I stopped contacts entirely because I was afraid, I could get infected. I was suddenly very lonely, yes.” (P19)
Emotional well-being, fear, and grief	Stress: “Retrospectively, it stressed me so much. It was terrible for me, and I feel like I haven’t had a vacation for a whole year, even though I worked less than normal. I feel drained because these 6 weeks of corona unemployment haven’t been a vacation for me.” (P14) “My blood sugar increased because of my fiancé [who could not enter Switzerland], because I have been very stressed about it for the last few months. I am currently taking metformin, and then I had to double the dose. My blood pressure has gone up with weight gain.” (P12) “It’s just that sometimes I have to work non-stop for 4 to 5 h on the screen and my children play behind it. It is not that easy… For a while, it was too much. I had to move my office desk, … I couldn’t work in peace. But for a family with children my age, maybe the parents are younger or can share responsibility and don’t have a risk like me with high blood pressure, atrial fibrillation, and overweight… I think my children have had to sacrifice a lot, more than others.” (P3) Fear: “I was afraid to go outside because you didn’t really know where you could get infected and how. It was all a big question mark.” (P1) “I’m afraid of corona. I have five children, and I am afraid that someone might have corona or my husband… I am stressed because I am terrified for my family.” (P4) “I was very afraid to meet other people because of my mother. That is why I was mostly at home. I did not see people. I missed it a lot… I got a bit depressed and also noticed that I was more frustrated. I also smoked more and generally I didn’t feel so well.” (P11) Depression: “The situation is not much different for me. I have always been at home a lot and have not done much because I am afraid of social contacts. But I became more depressed during the lockdown.” (P5) Grief: “I don’t have many friends in Switzerland and not a lot of contact to my family. My mother died during the pandemic, and I could not attend her funeral.” (P5)
Perspectives on health issues and risk for COVID-19 infection	Weight gain: “I must have gained 3 or 4 kg. The blood pressure is the same because I no longer have the stress of going to the office and coming home.” (P3) Identifying obesity as a risk: “I am 57, and therefore I don’t belong to the risk group yet. I do not have high blood pressure. I do not have diabetes, and therefore I do not see myself as a person at risk. I’m just overweight.” (P10) Doctors’ appointments: “No, I cancelled all my doctors’ appointments.” (P2) “I cancelled individual appointments, and I was able to do others over the phone. For example, nutritional consultations. I’m also consulting a psychologist about my eating habits. We were able to discuss these 2 to 3 times over the phone.” (P3) Lack of communication and support through healthcare providers “I suspended my family doctor during Corona. … in August … I was admitted to the hospital with a high fever, and it was assumed that I had pneumonia and was hospitalized to the corona ward. With a fever of 40 degrees in the university hospital, they believe Corona in this situation. The university hospital tried to contact my doctor, but they didn’t reach him for four days, and I think that’s very bad.” (P17)
